# Evidence of Specialized Tissue in Human Interatrial Septum: Histological, Immunohistochemical and Ultrastructural Findings

**DOI:** 10.1371/journal.pone.0113343

**Published:** 2014-11-20

**Authors:** Lubov B. Mitrofanova, Andrey N. Gorshkov, Dmitry S. Lebedev, Evgeny N. Mikhaylov

**Affiliations:** 1 Department of Pathology, Federal Almazov Medical Research Centre, Saint-Petersburg, Russia; 2 Laboratory of cell morphology, Institute of cytology of the Russian Academy of Sciences, Saint-Petersburg, Russia; 3 Laboratory of structural and functional proteomics, Research Institute of Influenza, Saint-Petersburg, Russia; 4 Department of Arrythmology, Federal Almazov Medical Research Centre, Saint-Petersburg, Russia; 5 Neuromodulation unit, Federal Almazov Medical Research Centre, Saint-Petersburg, Russia; Gent University, Belgium

## Abstract

**Background:**

There is a paucity of information on structural organization of muscular bundles in the interatrial septum (IAS). The aim was to investigate histologic and ultrastructural organization of muscular bundles in human IAS, including fossa ovalis (FO) and flap valve.

**Methods:**

Macroscopic and light microscopy evaluations of IAS were performed from postmortem studies of 40 patients. Twenty three IAS specimens underwent serial transverse sectioning, and 17 - longitudinal sectioning. The transverse sections from 10 patients were immunolabeled for HCN4, Caveolin3 and Connexin43. IAS specimens from 6 other patients underwent electron microscopy.

**Results:**

In all IAS specimens sections the FO, its rims and the flap valve had muscle fibers consisting of working cardiac myocytes. Besides the typical cardiomyocytes there were unusual cells: tortuous and horseshoe-shaped intertangled myocytes, small and large rounded myocytes with pale cytoplasm. The cells were aggregated in a definite structure in 38 (95%) cases, which was surrounded by fibro-fatty tissue. The height of the structure on transverse sections positively correlated with age (P = 0.03) and AF history (P = 0.045). Immunohistochemistry showed positive staining of the cells for HCN4 and Caveolin3. Electron microscopy identified cells with characteristics similar to electrical conduction cells.

**Conclusions:**

Specialized conduction cells in human IAS have been identified, specifically in the FO and its flap valve. The cells are aggregated in a structure, which is surrounded by fibrous and fatty tissue. Further investigations are warranted to explore electrophysiological characteristics of this structure.

## Introduction

Structural organization of interatrial septum (IAS) has been extensively investigated in animals and humans; it has been noted that in fossa ovalis (FO) and its flap valve there are muscular bundles, participating in interatrial electrical conduction [1]. Furthermore, IAS has been recognized as a source of focal and reentrant atrial tachycardias [2], [3]. However, there is a paucity of information on organization and fine structure of muscular bundles and myocardial cells in the IAS. We hypothesized, that the IAS muscular bundles might have special organization, different from regular contraction myocardium, in respect that the IAS has complex nature and plays an important role in interatrial conduction.

The aim of our study was to investigate histologic organization of muscular bundles in the human IAS (including FO and flap valve) with the use of immunohistochemical markers for the specialized conduction tissue, and to evaluate myocardial cells in this area using electron microscopy.

For immunohistochemical labeling we have chosen the following markers: a non-selective marker (Caveolin3), confirming that the investigated cells are myocytes; Connexin43, demonstrating gap junctions in working myocytes; and HCN4, the major isoform of the funny channel, more specific for specialized pacemaker and conduction cells.

## Methods

### Autopsy subjects

All autopsies were performed in the Almazov Centre and in the Bureau of Forensic Medicine (Saint-Petersburg). Participant's next of kin provided written informed consent to this study. The study and informed consents were approved by the Almazov Centre local ethics committee. Light microscopy evaluations of the IAS were carried out from postmortem studies of 40 patients; immunohistochemical labeling was performed in 10 of these patients; additional IAS specimens from 6 other patients underwent electron microscopy. Thus, IAS evaluations were carried out from a total number of 46 patients (characteristics are present in Table 1).

**Table 1:** Characteristics of the patients and histological material.

		Overall	Light microscopy		Electron microscopy
			Longitudinal sections	Transverse sections	
**N**		46	17	23	6
**Age**		56.0±13.7	57.1±16.5	57.8±13.3	53.0±11.2
**Sex, M%**		29 (63.0%)	12 (70.6%)	13 (56.5%)	4 (66.7%)
**AF**	**Paroxysmal**	9 (19.6%)	6 (35.3%)	2 (8.7%)	1 (16.7%)
	**Chronic**	9 (19.6%)	3 (17.6%)	6 (26.1%)	0
**CAD**		23 (50%)	8 (47.1%)	13 (56.5%)	2 (33.3%)
**Mitral valve disease**		4 (8.7%)	2 (11.8%)	2 (8.7%)	0
**Aortic valve disease**		2 (4.3%)	0	2 (8.7%)	0
**GI cancer**		7 (17.2%)	2 (11.8%)	2 (8.7%)	3 (50%)
**COPD**		1 (2.2%)	1 (5.9%)	0	0
**DCM**		3 (13.0%)	2 (11.8%)	1 (4.3%)	0
**LV noncompaction (heart transplant)**		1 (2.2%)	0	0	1 (16.7%)
**No known disease**		6 (13.0%)	3 (17.6%)	3 (13.0%)	0
**Causes of death**	**Asphyxia (violent death)**	6 (13.0%)	2 (11.8%)	4 (17.3%)	0
	**Myocardial infarction**	21 (45.7%)	8 (47.1%)	11 (47.8%)	2 (33.3%)
	**Stroke**	5 (10.9%)	2 (11.8%)	3 (13.0%)	0
	**Pulmonary embolism**	6 (13.0%)	4 (23.5%)	2 (8.7%)	0
	**Cancer**	4 (8.7%)	1 (5.9%)	3 (13.0%)	0
	**Pneumonia**	3 (6.5%)	0	0	3 (50%)
**Conduction-like cardiac cells**		46 (100%)	17 (100%)	23 (100%)	6 (100%)
**Aggregation of conduction-like cells into a structure**		44 (95.6%)	17 (100%)	21 (91.3%)	6 (100%)
**Fibrous and fatty capsule around conduction-like cells**		44 (95.6%)	17 (100%)	21 (91.3%)	6 (100%)

AF, atrial fibrillation; CAD, coronary artery disease; GI, gastrointestinal; COPD, chronic obstructive pulmonary disease; DCM, dilated cardiomyopathy; LV, left ventricle. P>0.05 for all parameters.

**Figure 1:**
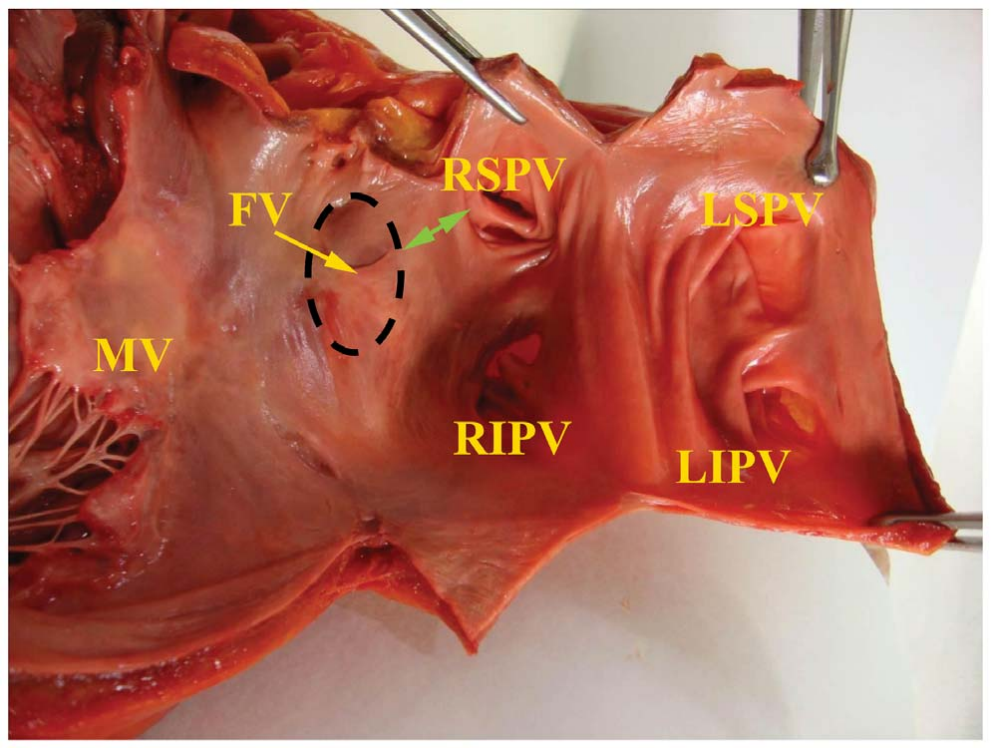
Left atrial endocardial surface with an IAS and a part of the mitral valve. The heart obtained from a 55-year-old male patient. FO, fossa ovalis; FV, flap valve; RSVP, right superior pulmonary vein ostium; RIPV, right inferior pulmonary vein ostium; LSVP, left superior pulmonary vein ostium; LIVP, left inferior pulmonary vein ostium; MV, mitral valve. The distance between the RSPV and the flap valve is marked by the green arrow. The dotted oval denotes the most frequently encountered location of the structure.

Macroscopic study of IAS followed a conventional autopsy protocol and included the following measurements: FO size, flap valve length, distances between anatomical structures (right superior pulmonary vein (RSPV), FO, flap valve, mitral annulus, atrioventricular (AV) node). IASs were excised from hearts and underwent further evaluation. The IAS specimens included: a FO with its rims, a flap valve of the fossa, an ostium of the RSPV (**Figure 1**).

### Light microscopy

IASs were excised from free atrial walls, fixed in 10% buffered formalin and embedded in paraffin blocks. The IAS specimens from 23 patients underwent serial transverse sectioning, which was directed from a posterior to an anterior part with 1 mm steps, starting from the most superior point of the IAS and going down to the AV node. The sectioning was performed parallel to the mitral annulus. This technique has been previously described in detail [4].

The IAS specimens from 17 patients underwent longitudinal sectioning. A part of the RSPV (5 mm length) was left in specimens. The paraffin blocks with IASs were adjusted to the IAS thickness, and sectioned into about 15 slices each (slice thickness 3 µm); the sectioning was performed parallel to the endocardial surface [4].

All obtained transverse and longitudinal sections were stained with hematoxylin-eosin, van Gieson stain or with Masson's trichrome stain, and then were evaluated by light microscopy with morphometric analysis of cells and cell patterns using a computer-assisted morphometric Leica LAS Image Analysis System (LeicaQWin Plus v3, Leica Microsystems IS, Cambridge, UK).

Dimensions of structures on the transverse sections were calculated from the number of consecutive parallel slides with a known distance (1 mm) between them.

### 3-dimensional reconstruction

A three-dimensional reconstruction of an IAS was performed on a specimen after serial transverse sectioning (107 sections) and staining with hematoxylin-eosin. Structures on the sections were manually marked under a light microscope, and a 3D model was reconstructed using the Free-D 1.10 software (INRA, France; [5]).

### Immunohistochemistry

Immunohistochemical methods were applied to the transverse IAS sections, obtained from 10 patients and embedded in paraffin. We assessed the distribution of hyperpolarization-activated and cyclic nucleotide–gated channel 4 (HCN4), Connexin43 and Caveolin3. Paraffin embedded fixed material was first deparaffinized/rehydrated using Xylol and descending series of Ethanol solutions. Then the sections were pre-treated with 3% hydrogen peroxide (room temperature, 5 min) in order to inactivate endogenous peroxidase. Then the sections were washed in distilled water. An antigen retrieval procedure was carried out in Tris-EDTA (pH 9.0) at 95–98°C for 35 min (TRS 9.0, Dako, Denmark). Then the material was cooled at room temperature. Subsequently the sections were washed in Tris-Buffered Saline with Tween 20 two times for 5 min (TBS, Dako, Denmark). The mounted sections were circumscribed with a hydrophobic pen (Elite PAP Pen, Diagnostic BioSystems, USA). Immunolabeling was performed with the following antibodies: a mouse monoclonal IgG against HCN4 at a dilution 1∶50 (clone S114-10, Abcam, UK), and a rabbit polyclonal antibody against HCN4 (Alamone Labs, Jerusalem, Israel); a rabbit polyclonal IgG against Connexin43 at a dilution 1∶50 (Diagnostic BioSystems, USA) and a mouse monoclonal IgG against Caveolin3 at a dilution 1∶50 (clone C-2; Santa Cruze Biotechnology, USA). After incubation with primary antibodies the sections were two times washed in Tris-Buffered Saline with Tween 20. For detection of the primary antibodies we used the Real EnVision Detection System, Peroxidase/DAB, Rabbit/Mouse kit (DAKO, Denmark). Incubation with the primary and secondary antibodies was performed for 30 min at room temperature. After washing in distilled water the sections were counterstained with Hematoxylin (2 min), dehydrated, and then coverslips were mounted using permanent mounting medium (Polystyrol, BioMount, Italy).

For a double immunolabeling the following steps were performed. Paraffin tissue sections were deparaffinized, and then washed 2 times in Tris-Buffered Saline with Tween 20. Tissue pretreatment was performed with heat-induced epitope retrieval – Tris-EDTA (pH 9.0) at 95–98°C for 35 min, followed by cooling at room temperature for 20 min. Ultra V Block solution was applied with subsequent incubation for 10 min at room temperature to block nonspecific background staining. A primary antibody cocktail (#1 or #2) was applied, and incubation for 30 min at room temperature was performed. A primary antibody cocktail #1 consisted of a mouse monoclonal IgG against HCN4 at a dilution 1∶50 (clone S114-10, Abcam) and a rabbit polyclonal IgG against Connexin43 at a dilution 1∶100 (Cell Signaling Technology, USA). A primary antibody cocktail #2 consisted of a rabbit polyclonal IgG against HCN4 at a dilution 1∶200 (Alamone Labs) and a mouse monoclonal IgG against Caveolin3 at a dilution 1∶50 (clone C-2, Santa Cruze Biotechnology). The material was incubated with MultiVision Polymer Cocktail (Thermo Scientific, UK): anti-rabbit/HRP + anti-mouse/AP for 30 min at room temperature. Working solutions of LVBlue and LVRRed were applied and incubated for 10 min each. After final washing tissue sections were dried and embedded into glycerin. When the cocktail #1 was applied, a blue marker showed HCN4 protein, and a red marker – Connexin43. When the cocktail #2 was applied, a blue marker showed Caveolin3, and a red marker – HCN4.

The double immunolabeling HCN4-Connexin43 was applied to two specimens; the HCN4-Caveolin3 double immunolabeling was applied to three specimens. Specimens from another heart were used for control immunolabeling of sinus node cells and working myocytes. A co-localization coefficient between two proteins was calculated under light microscope and using an image analyzer ImageScope Color M (Russia).

### Electron microscopy

Electron microscopy was performed on IAS specimens obtained from 6 patients (Table 1). The material for electron microscopy was fixed in 5 minutes following excision of the recipient's heart in one case and after 1–2 hours of death in other patients.

The IAS specimens were cut in 10–15 small pieces (1–2 mm in diameter) each, which were pre-fixed in 2.5% solution of glutaraldehyde on 0.1 M phosphate buffer (pH 7.4) for 45 min at room temperature, then the pieces were three times washed in phosphate buffer and post-fixed in 1% solution of OsO_4_ on the same buffer for 1 hour. Then the specimens were dehydrated in series of ethanol solutions of gradually increasing concentration and embedded in the Epon epoxy resin.

The semi-thin (1–2 µm) sections were obtained from the embedded specimens. The sections were stained with toluidine blue and analyzed under the light microscope. The sections containing “unusual” myocytes (those different from usual working cardiac myocytes) were selected for subsequent electron microscopy. Thus, at least 4 blocks from each IAS underwent ultrathin sectioning (70–80 nm) using a ultramicrotome (LKB, Sweden). The sections were collected on the copper grids for the electron microscopy and contrasted with uranyl-acetate and lead citrate. Analysis of sections was performed using transmission electron microscopes Libra 120 (Carl Zeiss, Germany) and JEM 1011 (JEOL, Japan).

### Statistical analysis

Associations between categorical and non-normally distributed variables were tested with Fisher's exact test; continuous variables – with Student's t-test. A 2-tailed P<0.05 indicated statistical significance. Correlations for normally distributed variables were tested using linear Pearson correlation test; for non-normally distributed variables Spearman test was used. Analysis was performed using Statistica 6.0 (StatSoft Inc., USA).

## Results

### Macroscopic study

The mean heart weight was 445.3±70.2 g. The mean FO size was 17.6±7.9×16.7±5.9 mm. The mean distance between the RSPV and the FO flap valve was 21.8±6.8 mm. The flap valve had a semilunar shape in all cases (**Figure 1**) with a mean length of 25.2±11.3 mm.

### Light microscopy of transverse IAS sections

**Figure 2:**
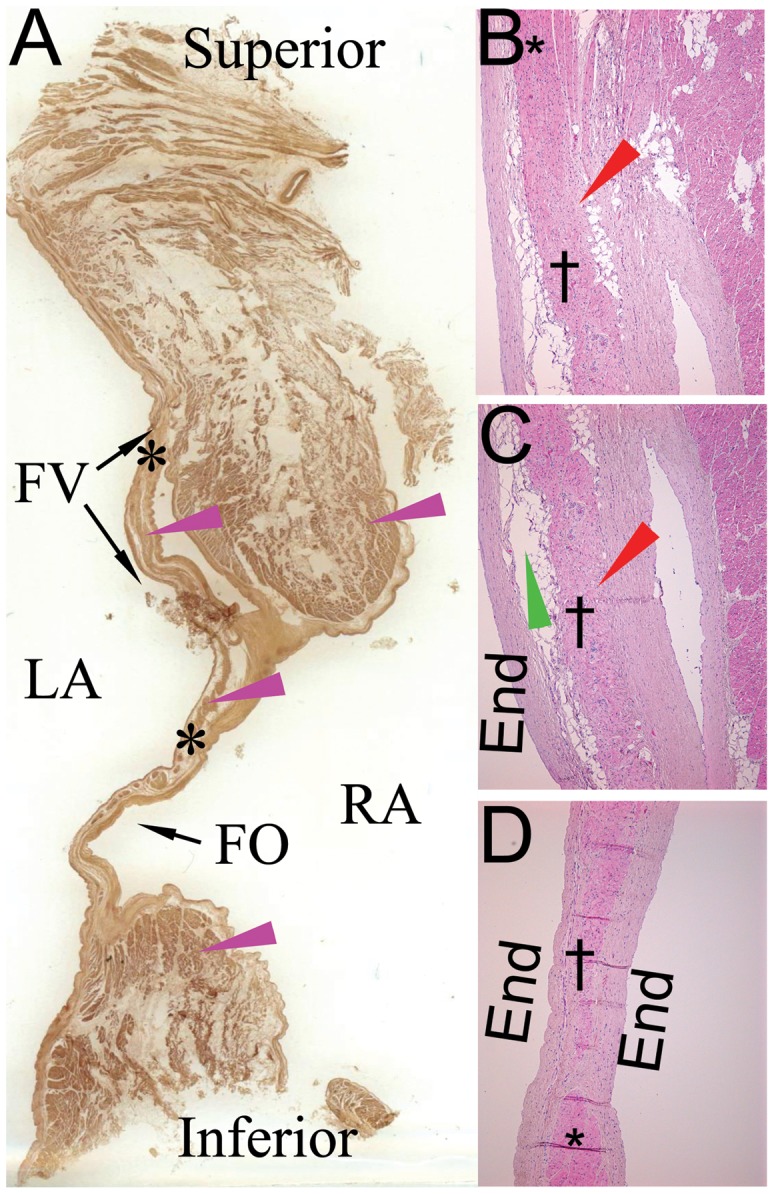
An IAS with the flap valve. The heart obtained from a heart of a 62-year-old female patient. **A**: A transverse section of the IAS. FO, fossa ovalis; FV, flap valve; LA, left atrium; RA, right atrium. Muscle fibers are shown by purple arrowheads. Black asterisks denote localization of the structure. **B, C and D**: Light micrographs of consecutive sections of the flap valve; (hematoxylin-eosin; ×50). The sections performed along the flap valve form its basis (A) to the middle part (B), and distally at the central part of the FO. **B.** Atypical myocytes (specialized-like) are in direct contact with contraction myocardium in the upper part of the interatrial septum. **C and D.** Along the further course of the structure, it is surrounded by fibrous and fatty tissue. *Typical working cardiomyocytes; †atypical specialized-like myocytes; End, endocardium; fibrous tissue is marked by red arrowheads, fatty tissue is marked by green arrowheads.

**Figure 3:**
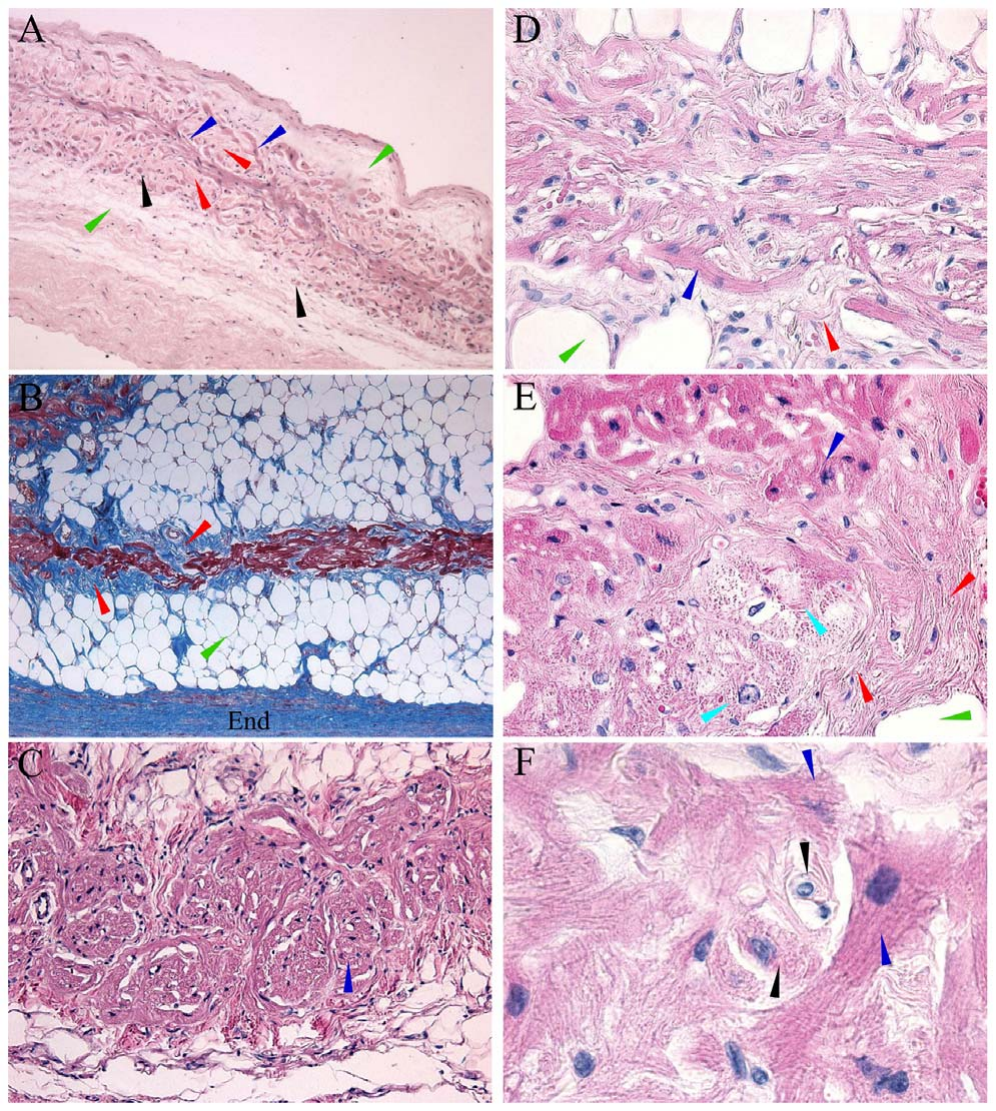
Light micrographs of transverse IAS sections. The heart obtained from a 62-year-old male patient. T-like cells (blue arrowheads), P-like cells (black arrowheads), Purkinje-like cells (turquoise arrowheads). Aggregated T-like cells are surrounded by fibrous tissue (red arrowheads) and then by fat (green arrowheads). End, left atrial endocardium. **A.** T-like cells, P-like cells, fibrous and fat tissues in the FO (hematoxylin-eosin; ×100). **B.** The structure consisting of T-like cells surrounded by fibrous and fatty tissue in the FO flap valve (Masson's trichrome; ×100). **C.** Tortuous, horseshoe-shaped T-like cells forming fan-shaped figures in the FO. (hematoxylin-eosin; ×200). **D.** T-like cells with side-end contacts (the blue arrowhead) (hematoxylin-eosin; ×400). **E.** Purkinje-like cells (turquoise arrowheads) are surrounded by T-like cells, fibrous and fatty tissue (hematoxylin-eosin; ×400). **F.** T-like and P-like cells in the FO flap valve (hematoxylin-eosin; ×1000).

In all 23 (100%) specimens the FO, its rims and the flap valve had muscle fibers consisting of contraction cardiac myocytes (**Figure 2a, b**). Immediately below the endocardium among typical contraction myocytes in the muscular bundles, in the flap valve, antero-superior FO rim, and in the FO, there were tortuous and horseshoe-shaped intertangled myocytes that formed fan-shaped, X-shaped, Y-shaped, and other figures. These cells were found in all 23 (100%) cases, and they were aggregated into a definite structure in 21 (91%) cases (Table 1). The structure was extended from the flap valve to the FO, and to its antero-superior rim. The tortuous myocytes had a mean diameter of 12.4±4.3 (8.4–16.0) µm, and a length of 40–250 µm (**Figure 3**). In the centre of this structure myocytes were shorter, and on the periphery they were longer. Besides this type of cells, there were small (8.1±3.3 (5.2–10.0) µm) rounded myocytes with pale cytoplasm, and large (28.5±11.7 (25.5–40.0) µm) rounded myocytes with pale cytoplasm. According to their histological characteristics the myocytes were similar to specialized conduction cells: (1) the tortuous myocytes - like T-cells; (2) the small rounded cells with pale cytoplasm - like pacemaker P-cells of the sinoatrial and AV nodes; (3) the large pale myocytes – like Purkinje cells (**Figure 3**).

The discovered structure with the tortuous cells contained vessels and nerves. The cells of the structure directly contacted with contractile myocardium in the upper part of the septum (**Figure 2b**); and then along their further course to the FO, the cells were surrounded by fibrous tissue of coarse collagen fibers and fatty tissue (**Figure 2c, d**). The mean dimensions of the structure were the following: length 7.4±3.3 mm, height 10.2±8.0 mm, thickness 0.4±0.2 mm. In 11 (47%) patients the structure was located in the anterior part of the FO (closer to the mitral annulus), in 4 (17%) patients – in the superior part, in 8 (36%) it was extended to the anterior and superior parts. The mean distance between the structure and the AV-node was 15.4±13.2 mm.

In 2 (9%) cases the fibro-fatty capsule was not continuous, but the myocytes had the same characteristics.

A detailed description of the size and location of clusters (structures) with specialized-like cells in each heart can be found in the **Table S1**.

### Light microscopy of longitudinal IAS sections

On the longitudinal IAS sections there were muscular fibers crossing the FO and its flap valve. Tortuous, small rounded and large myocytes with pale cytoplasm were found in all specimens (Table 1). As on the transverse sections, the cells formed fan-shaped and other figures, and were aggregated in a structure, surrounded by fibrous tissue with vessels and nerves and surrounded by fatty tissue. The mean distance between the RSPV ostium and the structure was 12.5±5.9 (8–35) mm. The tortuous myocytes had the same dimensions as on transverse sections, and were in contact with contraction myocytes. The working myocytes were oriented horizontally to the structure in the forward direction, and at a sharp angle toward the infero-posterior rim of the fossa. Detailed data obtained on the longitudinal sections can be found in the **Table S2.**

No association was found between the sizes of the conduction-like cells, the presence of fibrous capsule around the structure, location of the structure in the FO and the history of atrial fibrillation or presence and type of structural heart disease. Although no association between the volume of the structure and any other parameter, the height of the structure on transverse sections positively correlated with AF history (P = 0.045) and age (P = 0.03). The presence of structural heart disease was associated with a longer distance between the structure and the AV node; however, structural disease was also positively associated with increased heart weight (P = 0.046) and the distance between the right superior pulmonary vein ostium and the flap valve (P = 0.019).

**Figure 4:**
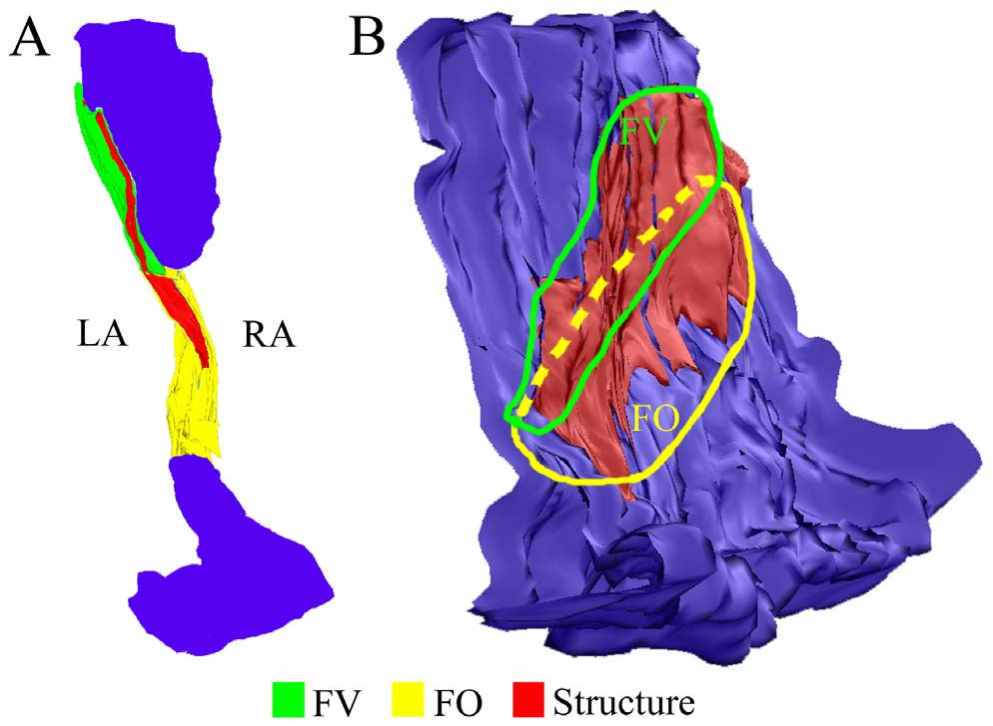
A 3-dimensional reconstruction of a specimen containing an IAS. The reconstruction performed from 107 serial sections of the IAS (obtained from a 50-year old male patient) demonstrates spatial orientation of the structure with the specialized-like myocytes. **A:** A model of the IAS transverse section (shown as in **Figure 2A**). **B:** A model of the IAS specimen, view from the left atrial side. Red color represents the structure with the specialized-like myocytes. Yellow color represents the fossa ovalis (FO). Green color represents the flap valve (FV). LA, left atrial side; RA, right atrial side.

A three-dimensional reconstruction of serial IAS histological sections representing correlative localization of the structure with the specialized-like cells is shown in **Figure 4**.

### Immunohistochemistry

**Figure 5:**
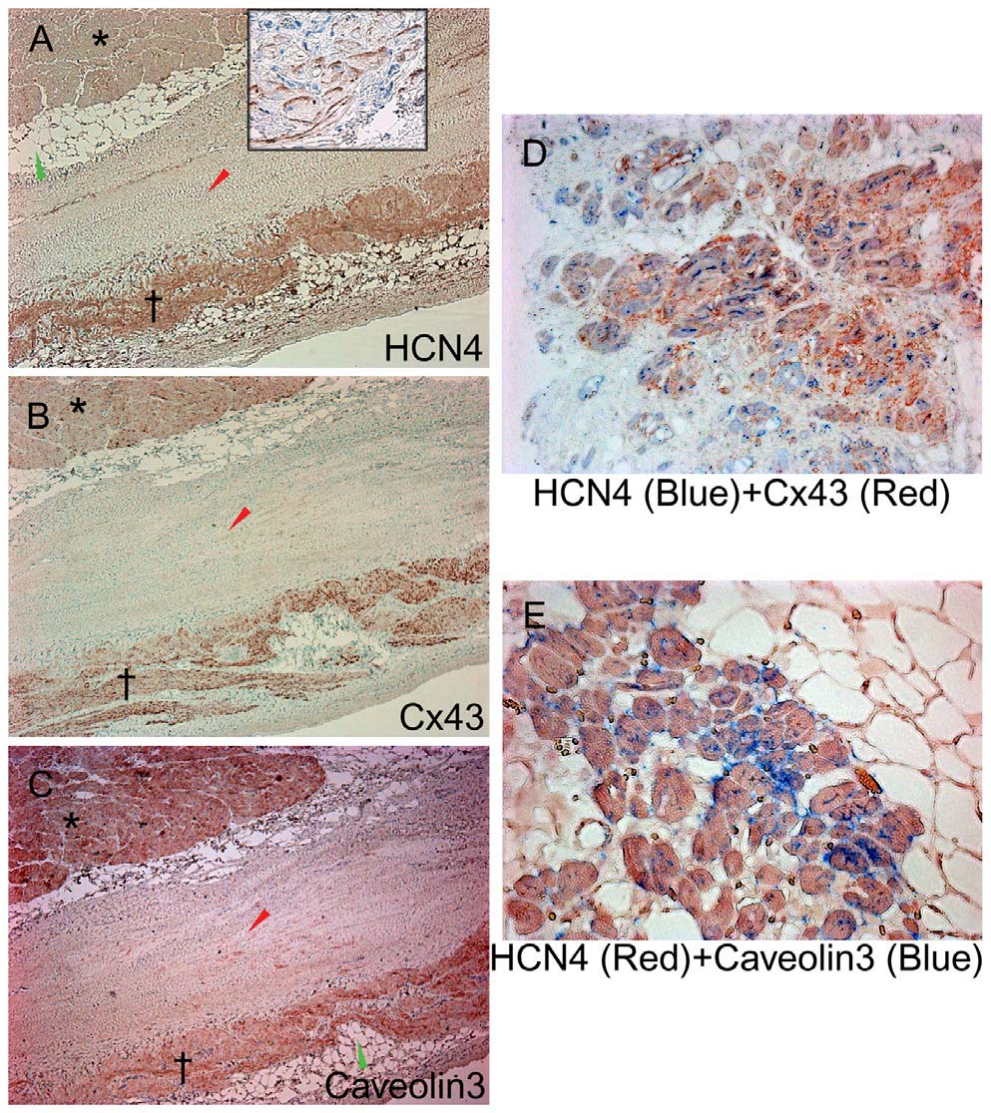
Immunohistochemical labeling of serial transverse IAS sections. **A.** Labeling for HCN4 (the heart obtained from a 58-year old male patient; brown color; a mouse monoclonal antibody, Alamone Labs, Israel; hematoxylin counterstain; ×50). Positive labeling of the specialized-like cells (†), and negative labeling of the typical contraction myocytes (*). A close up view shows sub-membrane immunolabeling with HCN4 of the T-like and P-like cells (a rabbit polyclonal antibody, Abcam, UK; hematoxylin counterstain; ×200). **B.** Labeling for Connexin43 (Cx43) (the heart obtained from a 58-year old male patient; brown color; hematoxylin counterstain; ×50). Notably, positive staining of the specialized-like cells, as well as of typical contraction myocytes, was found in 2 specimens. **C.** Labeling for Caveolin3 (brown color; hematoxylin counterstain; ×50). **D.** Double immunolabeling (primary antibody cocktail #1) for HCN4 (blue color, Abcam, UK) and Connexin43 (Cx43) (red color) showing co-localization of the proteins. The heart obtained from a 63-year old female patient; ×100. A co-localization coefficient HCN4:Cx43 = 1∶3. E. Double immunolabeling (primary antibody cocktail #2) for HCN4 (red color, Alamone Labs, Israel) and Caveolin3 (blue color) showing co-localization of the proteins (the heart obtained from a 63-year old female patient; ×400). A co-localization coefficient Caveolin3:HCN4 = 1∶4.

The tortuous, specialized-like myocytes, located in the FO, its valve and its rim were positively immunolabeled for HCN4 and Caveolin3 (**Figure 5a, c**). Sections from 8 out of 10 blocks were negative for Connexin43. Sections from 2 blocks showed positive labeling of specialized-like cells to Connexin43 (**Figure 5b**), similar to positive immunolabeling of the working myocytes. A double immunolabeling was performed in those two specimens to HCN4 and Connexin43 (**Figure 5d**). A co-localization coefficient HCN4:Connexin43 was 1∶3 (from 1∶2.7 to 1∶3.2) in the specialized-like myocytes, what was similar to sinus node control (**Figure S4**). A double immunolabeling to HCN4 and Caveolin3 is shown in **Figure 5e**; a co-localization coefficient Caveolin3:HCN4 was 1∶4.

Positive controls for immunolabeling to HCN4, Connexin43 and Caveolin3 to demonstrate a protein-specific “staining” in sinus node and working myocardium are present in **Figures S1, S2 and S3**, correspondingly. **Figure S4** shows a control double immunolabeling for HCN4 and Cx43 of specialized cells in the sinus node.

### Electron microscopy

Among usual contraction cardiac myocytes electron microscopy of the IAS specimens identified cells with ultrastructural characteristics similar to electrical conduction cells, previously described in the human sinoatrial, AV nodes and in the ventricles: T-, P-, and Purkinje-like cells [6], [7]. All three types of the cells were identified in all studied IAS specimens from 6 patients.

#### Transitional-like cells

**Figure 6:**
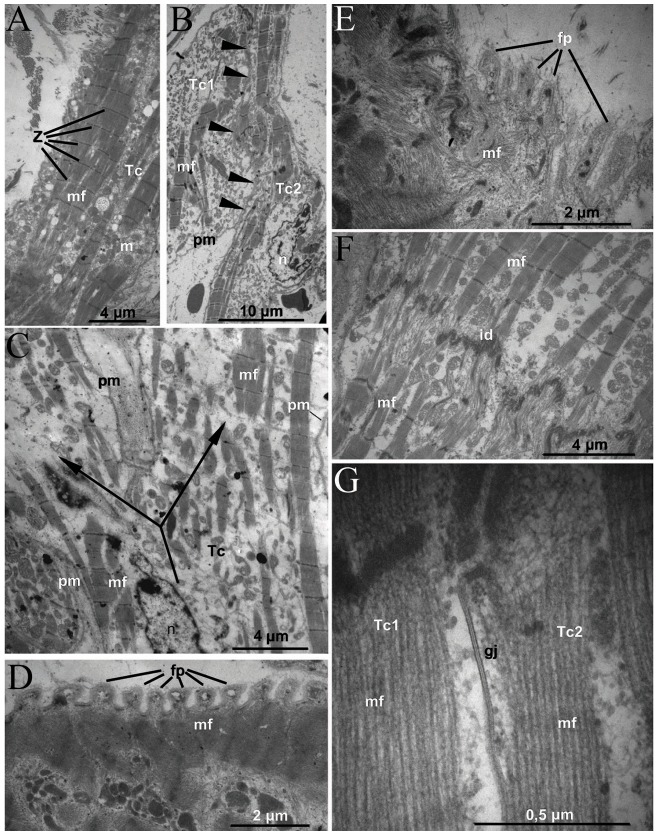
Electron micrographs of T-like cells in the IAS. The heart obtained from a 63-year-old female patient. Tc, T-like cell; mf, myofilaments; Z, Z-discs; m, mitochondria; n, nucleus; pm, plasma membrane; mf, myofilaments; fp – finger-shaped protrusions; id – intercalated disc; gj – gap junction. **A.** A general view of a T-like cell on the longitudinal section. **B.** Two bended apposed T-cells. Tc1, Tc2 – two T-cells, arrows indicate a boundary between two T-like cells. **C.** Bifurcation (arrows) of a T-cell body. **D and E.** Regular finger-shaped protrusions of the T-cell plasma membrane. **F.** An intercalated disc between two T-cells (“end to end” contact). **G.** A gap junction (nexus) between two T-cells (a “side-by-side” contact).

The T-like cells of the IAS had an elongated shape and diameter of 8–16 µm. Contractile material of the T-like cells was located predominantly in the peripheral part of the cytoplasm and had regular sarcomere structure (**Figure 6a**). There was no clear zonation of the T-like cells' cytoplasm. The myofilament bundles were sometimes found in a close proximity to a nucleus (**Figure 6b, c**). Areas of free from myofibrils cytoplasm were generally filled with numerous rounded or oval mitochondria. However, in contrast to the well-known structural organization of the contraction cardiomyocytes, no alternation of myofibril bundles with rows of mitochondria was observed in the T-like cells. Mutual distribution of these organelles was rather irregular.

The T-like cells often had irregular shape, and this feature distinguished them from other cardiac myocytes, and especially from the contraction myocardium. The T-like cells were frequently bended, Y-bifurcated; two closely located T-like cells were “pressed” together (**Figure 6b, c**).

Plasma membrane of the T-like cells had multiple regular finger-shaped protrusions and invaginations with closely associated myofilaments (**Figure 6d, e**). The T-like cells were often joined to each other via well-developed intercalated discs. The intercalated discs were present in the zones of end-to-end contacts of the T-like cells (**Figure 6f**), and also on their lateral surface (the “side-by-side” contacts) (**Figure 6g**).

#### P-like cells

**Figure 7:**
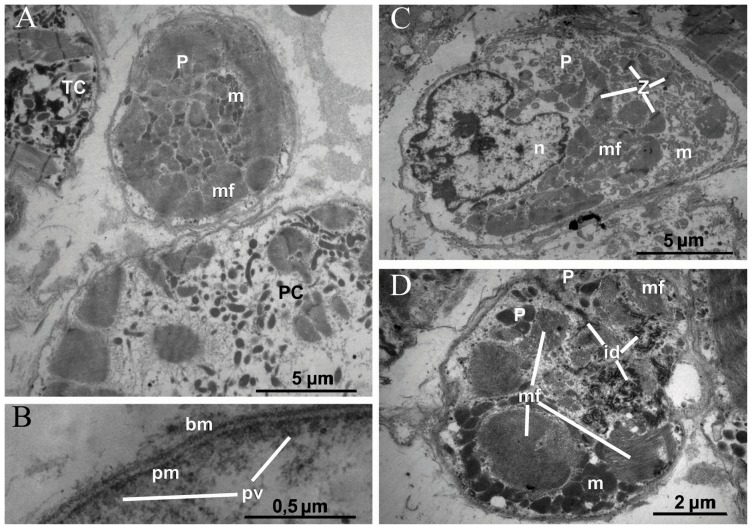
Electron micrographs of P-like cells in the IAS. The heart obtained from a 55-year-old male patient. P, P-like cell; PC, Purkinje-like cell; Tc, T-like cell; pm, plasma membrane; PV, pinocytic vesicles; bm, basal membrane; m, mitochondria; n, nucleus; pm, plasma membrane; mf, myofilaments; id – intercalated disc; Z, Z-discs. **A.** A round-shaped P-like cell neighboring a T-like cell and a Purkinje-like cell. **B.** Plasma membrane of a P-like cell forming multiple pinocytic vesicles and surrounded by the basal membrane. **C.** A P-like cell with numerous bundles of short myofilament which were cut at various angles. **D.** A cluster of two P-like cells joined via a complex-shaped intercalated disc.

The P-like cells were distributed among other cardiac myocytes of the IAS as single cells or as small clusters consisting of 2–3 cells. The P-like cells had quite regular rounded or slightly oval shapes, with no significant membrane invaginations. Their diameter ranged between 5 and 10 µm. (**Figure 7a, c, d**).

On the plasma membrane there were many pinocytic vesicles associated with the membrane. From the outside the membrane of the P-like cells, as well as of other cardiac myocytes, was covered with a basement membrane (**Figure 7b**). In cases, when these cells were present in a small group, the basement membrane covered the whole group.

Predominant organelles in the cytoplasm of the P-like cells were rounded mitochondria and short bundles of myofilaments. The myofilament system in the cytoplasm of the IAS P-like cells did not have such regular structure as in the contraction myocytes or in the T-cells. Particularly, myofilament bundles of the P-like cells had no regular sarcomere organization. In a single bundle only 1–2 Z-discs were present. Moreover, myofilaments were found with different angle alignment with respect to plasma membrane. Therefore, an ultrathin section of a single cell contained parallel and transversely cut myofilament bundles (**Figure 7c**). This morphologic feature allowed to easily distinguish the P-like cells from other transversely cut myocytes of similar size (especially T-cells processes).

In cases when several P-like cells formed a cluster, there was an intercalated disc within the cluster. Components of intercalated discs were very tortuous, suggesting high complexity of a three-dimensional disc structure (**Figure 7d**).

#### Purkinje-like cells

**Figure 8:**
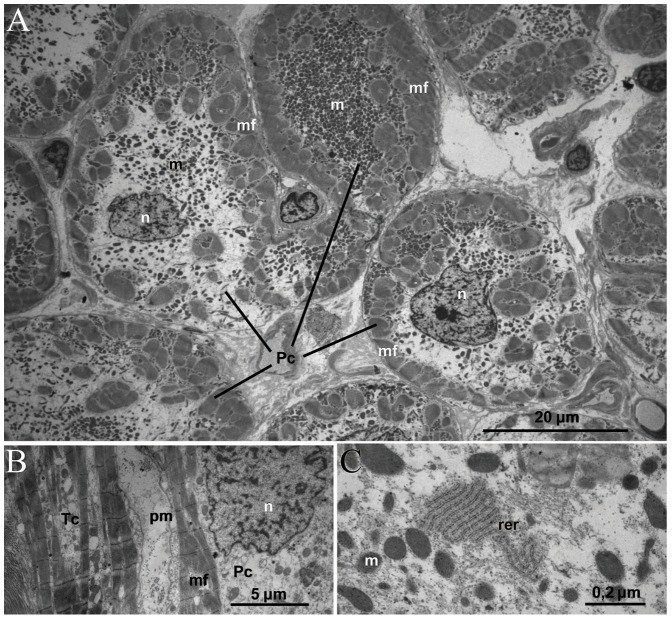
Electron micrographs of Purkinje-like cells in the IAS. The heart obtained from the same patient as in figure 6. Pc, Purkinje-like cell; n, nucleus; m, mitochondria; mf, myofilaments; pm, plasma membrane; rer - rough endoplasmic reticulum. **A.** A transverse section of several Purkinje-like cells. The large size of cells and localization of myofilaments at the cell periphery are appreciated. **B.** A longitudinal section of a Purkinje-like cell. The regular sarcomeric organization of myofilaments and plasma membrane undulations are visible. A T-like cell is also seen to the left from the Purkinje-like cell. **C.** Densely packed rough endoplasmic reticulum in Purkinje-like cell cytoplasm.

The Purkinje-like cells (**Figure 8**) were of a rounded or elongated shape with a 20–40 µm diameter in their central part, across the nucleus. Common morphologic feature of this type of cells in the IAS, along with their large size, was a quite clear zonation of the cytoplasm on a peripheral area, containing bundles of myofibrils, and an electron-light central perinuclear area free from myofibrils and full of numerous mitochondria (**Figure 8a, b**). Plasma membrane frequently had regular undulatory protrusions and invaginations (**Figure 8b**). Myofilament bundles of the Purkinje-like cells had predominant direction parallel to the long cell axis. On the longitudinal sections they had a regular sarcomere organization (**Figure 8b**). In the central area there were also densely packed cisternae of the rough endoplasmic reticulum (**Figure 8c**).

## Discussion

The major finding of the study is that within the human IAS cardiomyocytes with characteristics very close to specialized conduction cells have been identified. The cells are surrounded by fibro-fatty tissue, and are accompanied by nerves and vessels. Therefore, these cells are clearly aggregated in a distinct morphological structure. The cells within the structure permit the overall area to be distinguished from the adjacent tissue which can be morphologically followed from section to section. The specialized-like cells within the structure are in direct contact with working cardiomyocytes in the upper part of the IAS.

According to our findings, the structure was present in all hearts obtained from adult patients; on transverse sections the structure height positively correlated with the history of AF and age. An increased heart mass and structural heart disease was associated with a longer distance between the structure and the AV node. Whether this structure is present in fetuses or develops during ageing, needs further investigation.

### Immunohistochemical evaluation of the cardiac specialized cells

Specific immunohistochemical markers for the differentiation of specialized cells in the animal heart have been recently proposed and allowed to demonstrate the development of the human conduction system [8], [9]. These mainly include Connexins, transcription factors, funny current channel proteins, integral membrane proteins; and the most used of them are HCN4, Connexin43, Connexin40. Specificity of specialized cells detection using the immunohistochemical markers varies between different areas in the heart, and more comprehensive differentiation of the specialized tissue is believed to be the combination of immunohistochemical criteria and the old German criteria by Aschoff L and Mönckeberg JG [10].

In our study immunohistochemical labeling shows positive staining of the specialized cells for HCN4, the major isoform of the funny channel, along with labeling for Caveolin3, a scaffolding protein to concentrate within caveolar membranes of myocytes of different types.

According to our findings in eight cases, the structure with specialized-like cells has shown the lack of labeling for Connexin43, a marker of gap junctions; and positive labeling in two cases, which was very similar to working myocytes. This suggests that the specialized-like cells, localized in the septum, have gap junctions; thus, the cells might have transitional properties between specialized and working myocytes.

Double immunolabeling to HCN4 and Connexin43 in the specimens showing positive “staining” for both proteins, has revealed the co-localization coefficient very similar to that found in sinus node.

### Ultrastructural evaluation of the conduction system

The ultrastructural evaluation of the structure with the use of electron microscopy confirms the presence of specialized-like cells (T, P, Purkinje-like cells). Characteristics of the specialized cells have been extensively described in the past by James TN [11], [12], [13]. However, further research is this field has not been able to fully confirm the proposed ultrastructural criteria of conduction cells' definition in the human heart [6], [7].

One can stress out that ultrastructural findings in post-mortem human tissue are questionable when assessing criteria for specialization. In this regard, we suppose that the design of our study allowed preserving fine structures from significant postmortem changes or myolysis, since there was a very short period of time between death and material fixation for subsequent electron microscopy. Furthermore, within cytoplasm of the specialized-like cells normal organells with preserved structures (mitochondria, endoplasmatic reticulum, nuclei, intercellular junctions, etc.) present on the electron microscopy figures. Other cells of the IAS are present with normal shapes, sizes and organells' structures. It is difficult to assume that the presumed autolysis may lead to change in specialized-like cells' morphology, and induce no changes in other cells.

### Definition of the cardiac specialized tissue and controversies

The identified structure is partly in accordance with criteria of specialized conduction structures proposed in 1910 [14], [15], [16]: aggregated cardiac cells, histologically discrete from adjacent working myocardium; a structure consisting of the cells can be followed from section to section; a structure is insulated from the adjacent working myocardium by a sheath of fibrous tissue. The rules established by Aschoff L and Mönckeberg JG have been designed to arbitrate descriptions of tracts, and only the first two of the German criteria apply to the cardiac nodes. It should be noted that the structure that we have identified is not a “conduction tract”, and it is distant from both the AV and sinus nodes.

It might be suggested that with age the structure likely becomes more fibrous rather than muscular; therefore, the fibrous “surrounding” we have described might be as well the consequence of ageing. Thus, it cannot be certainly claimed that the described structure fulfills all criteria of the conduction tract; moreover, we have not identified distinct sites of origination and insertion of the structure, since it is completely located within FO and its rims.

Nevertheless, the combination of methods used in the study allows describing concentration of the specialized cells as a distinct structure.

The dimensions and composition of the structure is similar to those of the AV node [17]; however, the structure is nearly 1.5 cm away from the AV node, and can be located in the FO, the flap valve and the antero-superior rim of the FO.

As in the AV node, T-cells in the structure prevail over the others, and they have variable length and orientation [18].

Importantly, there is a serious controversial point regarding the presence of the Purkinje network in the human heart [19]. Findings in bovine hearts have suggested that there is a very small zone of transitional cells where the Purkinje fibers lose their connective tissue cover [20]; however, this has not been confirmed in other mammalians [21].

### Development of IAS and its flap valve

The IAS develops from two different regions. The septum primum, which embryologically grows from the roof of the primary atrial chamber, meets the so-called “septum secundum”, and this adhesion forms the IAS [22]. As it has been recently shown, the superior and posterior rims of the FO, along with much of the anterior rim, are only infoldings of the atrial walls [23]. Thus, the IAS consists of the floor of the FO, the flap valve, and the part of the antero-inferior rim of the fossa. The floor of the FO is the embryonic primary atrial septum, and the antero-inferior rim is produced by muscularisation of the vestibular spine. Thus, the true septum is made up of the flap valve of the oval foramen and its bulbous antero-inferior base.

From this perspective, the described structure is located in both the septum primum and the wall infoldings, formerly considered as the “septum secundum” [23]. The major part of the structure has been found in the true septum, with a mean distance from the RSPV of 12 mm.

### Conduction tissue in areas beyond sinoatrial and atrioventricular nodes

An extensive area within the terminal crest, near the sinus node, has been previously found [24]. The area represents a persisting part of primary myocardium [25].

Anderson RH and co-authors have shown that conduction structures exist within the vestibule of the tricuspid valve [26], and the specialized areas were sequestrated on the atrial side of the AV junction. In a human study a complete ring of immunohistochemically discrete tissue in the developing heart has been demonstrated [27]; currently, the ring is considered a remnant of extensive area of primary myocardium [28].

An additional node in retroaortic position has been found, and its existence has been recently confirmed by an immunohistochemical method [29].

One study results have suggested the presence of specialized cardiomyocytes within the human pulmonary venous sleeves [30]. In contrast, Ho SY and colleagues have subsequently shown, the myocytes in the sleeves are of working morphology, and no insulated specialized structure has been identified in this area [31]. Therefore, the substrate of pacemaking activity in this particular area has not been clarified yet.

An immunohistochemical study in rat hearts has shown a novel tract of nodal-like cells extending from the superior vena cava down the interatrial groove, and this area was capable of pacemaking [32].

Definitely, the structure found in our study is located in a different area, with no overlap with any previously investigated zone.

### Atrial arrhythmias and IAS

Potential association of the structure described in this study with atrial arrhythmias or interatrial conduction requires further investigation. Previous clinical studies have shown that focal atrial tachycardias can originate from the IAS, however, their incidence is not very high [33], [34]. Substrate of macro reentrant atrial tachycardias in patients without previous catheter ablation and surgical interventions has been also well-documented [3], [35]. The septal wall can be involved into bi-atrial reentrant atrial tachycardias [36].

The left septum has been recognized as a potentially arrhythmogenic site in patients undergoing catheter ablation for persistent atrial fibrillation. It is a common site for complex fractionated atrial electrograms and dominant frequency in atrial fibrillation [37], [38].

Ablation in the left IAS in patients with atrial fibrillation has been reported to result in organization to atrial tachycardia and/or restoration of sinus rhythm [37], [39], [40].

The presence of functional pacemakers in human IAS has been recently verified by both structural and functional examination of the human failing hearts [41]. The study demonstrated evidence of function pacemakers in region around AV junction which included IAS in heart failure patients.

Therefore, the septal wall can harbor focal, reentrant atrial tachycardias and substrate of atrial fibrillation.

## Limitations

A limitation of this study is that electrophysiological investigation of the IASs has not been performed; the study represents only anatomical, immunohistochemical and ultrastructural observations.

Another limitation is that we had no access to ECG and/or any electrophysiological evaluations in the majority of patients. Therefore, we are not able to provide comparative functional characteristics between patients with and without atrial tachyarrhythmias.

## Conclusions

The findings of our study suggest the presence of specialized conduction cells in the human IAS, specifically in the FO and its flap valve. The cells are aggregated in a structure, which is surrounded by fibrous tissue, and contains vessels and nerves. Further investigations are warranted to explore electrophysiological characteristics of this structure and its role in electrical conduction and/or atrial arrhythmias.

## Supporting Information

Figure S1 **Positive control for immunolabeling to HCN4.**
(DOC)

Figure S2 **Positive control for immunolabeling to Connexin43.**
(DOC)

Figure S3 **Positive control for immunolabeling to Caveolin3.**
(DOC)

Figure S4 **Positive control for double immunolabeling to HCN4 and Connexin43.**
(DOC)

Table S1 **Data obtained on interatrial septum transverse sections.**
(DOC)

Table S2 **Data obtained on interatrial septum longitudinal sections.**
(DOC)
