# Supplementary material for: Evidence of Specialized Tissue in Human Interatrial Septum: Histological, Immunohistochemical and Ultrastructural Findings
Source: PLoS One. 2014 Nov 20;9(11):e113343. doi: 10.1371/journal.pone.0113343 (PMC4239074; doi:10.1371/journal.pone.0113343)
Supplement: Figure S1 — Positive control for immunolabeling to HCN4. (DOC) [file pone.0113343.s001.doc]

**SUPPORTING FIGURE S1**


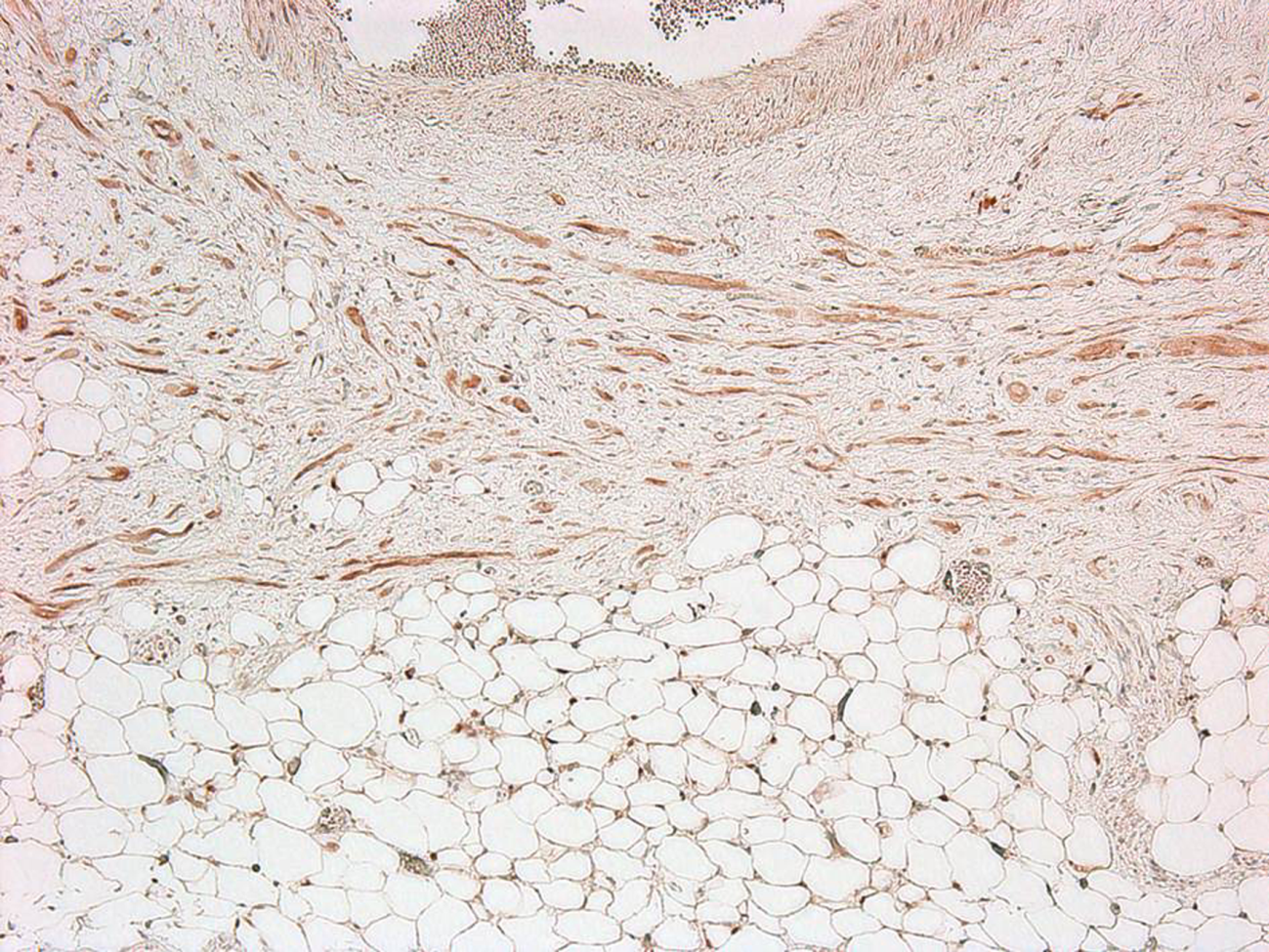


**Supporting figure S1. Positive control for immunolabeling to HCN4.**

Immunolabeling for HCN4 (brown color) of a sinus node section obtained from a 53-year old male patient (a rabbit polyclonal antibody, Alamone Labs, Israel; x100). A sinius node artery on the top of the figure; fatty tissue on the bottom.
