# Supplementary material for: Evidence of Specialized Tissue in Human Interatrial Septum: Histological, Immunohistochemical and Ultrastructural Findings
Source: PLoS One. 2014 Nov 20;9(11):e113343. doi: 10.1371/journal.pone.0113343 (PMC4239074; doi:10.1371/journal.pone.0113343)
Supplement: Figure S2 — Positive control for immunolabeling to Connexin43. (DOC) [file pone.0113343.s002.doc]

**SUPPORTING FIGURE S2**


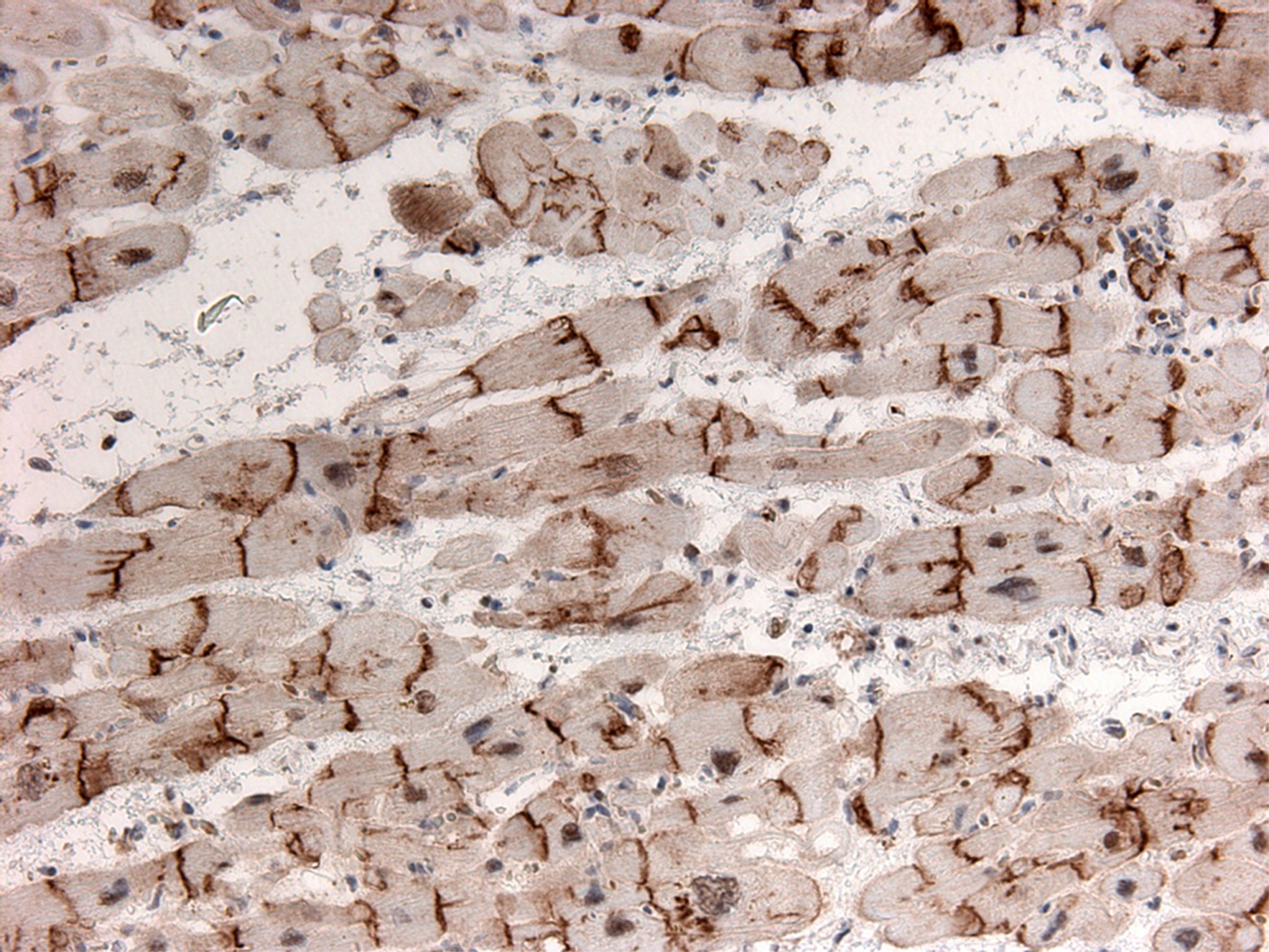


**Supporting figure S2. Positive control for immunolabeling to Connexin43.**

Immunohistochemical labeling for Connexin43 (brown color) of working myocardium obtained from a 53-year old male patient (a rabbit polyclonal antibody, Diagnostic BioSystems, USA; x200).
