# Supplementary material for: Evidence of Specialized Tissue in Human Interatrial Septum: Histological, Immunohistochemical and Ultrastructural Findings
Source: PLoS One. 2014 Nov 20;9(11):e113343. doi: 10.1371/journal.pone.0113343 (PMC4239074; doi:10.1371/journal.pone.0113343)
Supplement: Figure S3 — Positive control for immunolabeling to Caveolin3. (DOC) [file pone.0113343.s003.doc]

**SUPPORTING FIGURE S3**


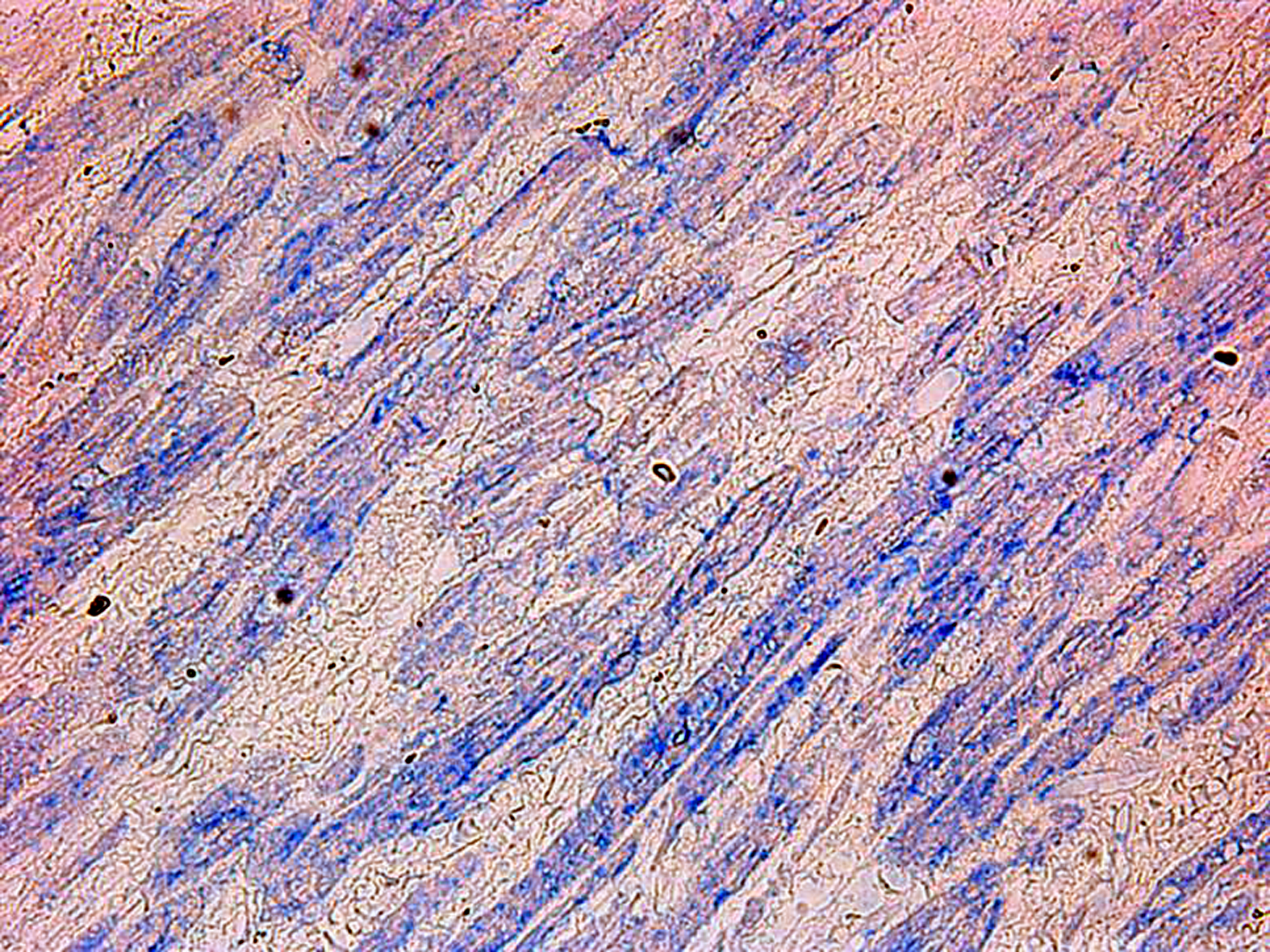


**Supporting figure S3. Positive control for immunolabeling to Caveolin3.**

Immunohistochemical labeling for Caveolin3 (blue color) of working myocytes obtained from a 53-year old male patient (a mouse monoclonal antibody, Santa Cruze Biotechnology, USA; x100).
