# Supplementary material for: Evidence of Specialized Tissue in Human Interatrial Septum: Histological, Immunohistochemical and Ultrastructural Findings
Source: PLoS One. 2014 Nov 20;9(11):e113343. doi: 10.1371/journal.pone.0113343 (PMC4239074; doi:10.1371/journal.pone.0113343)
Supplement: Figure S4 — Positive control for double immunolabeling to HCN4 and Connexin43. (DOC) [file pone.0113343.s004.doc]

**SUPPORTING FIGURE S4**


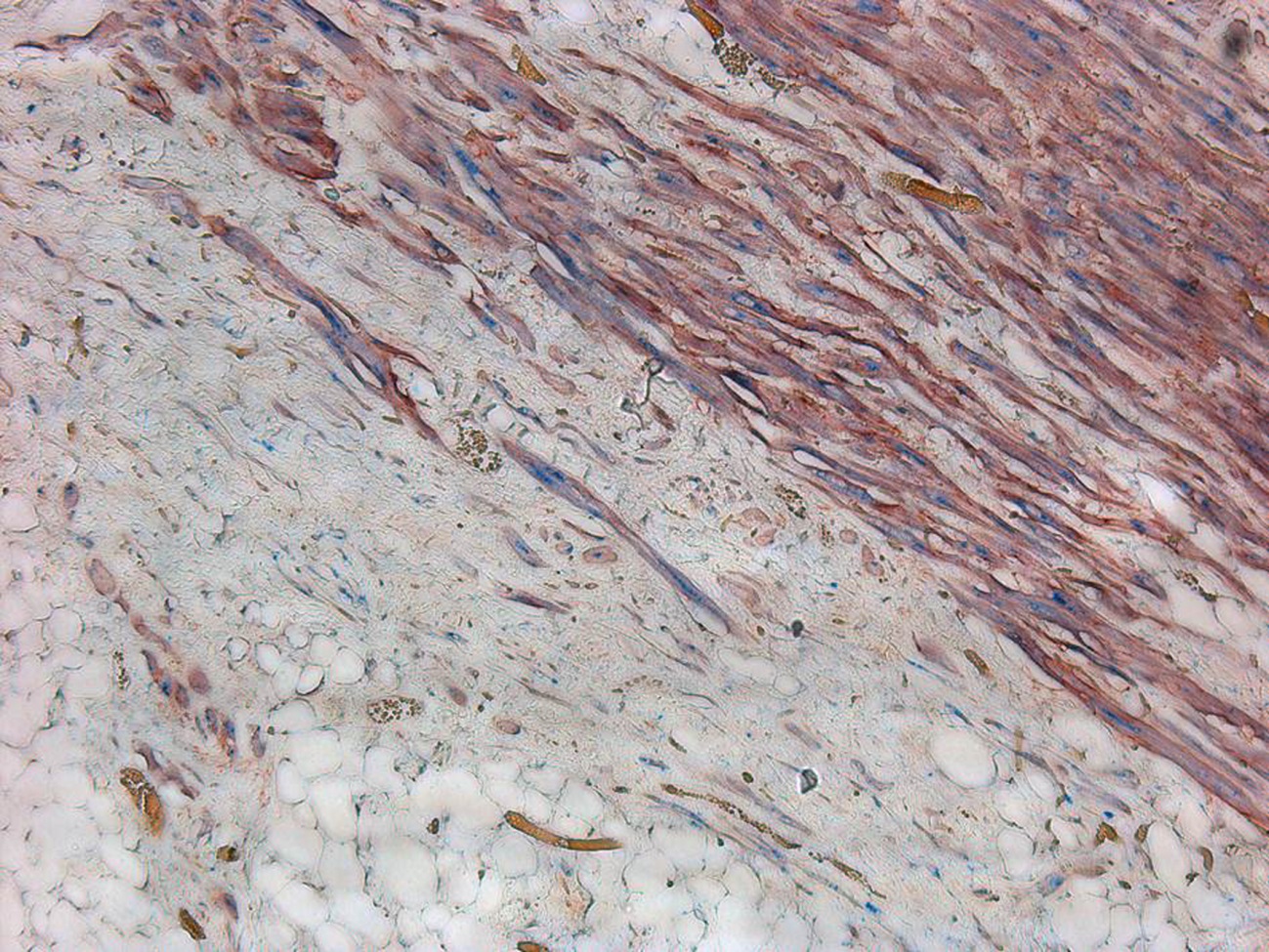


**Supporting figure S4. Positive control for double immunolabeling to HCN4 and Connexin43.**

A double immunolabeling for HCN4 (blue color, Abcam, UK) and Connexin43 (red color, Diagnostic BioSystems, USA) proteins demonstrating their co-localization in the sinus node cell. Sinus node myocytes on the top of the figure; connective and fatty tissue on the bottom (x100).

A co-localization coefficient for HCN4 and Caveolin3 is 1/3.
