# Supplementary material for: Evidence of Specialized Tissue in Human Interatrial Septum: Histological, Immunohistochemical and Ultrastructural Findings
Source: PLoS One. 2014 Nov 20;9(11):e113343. doi: 10.1371/journal.pone.0113343 (PMC4239074; doi:10.1371/journal.pone.0113343)
Supplement: Table S1 — Data obtained on interatrial septum transverse sections. (DOC) [file pone.0113343.s005.doc]

**Supporting TABLE S1**

Individual data regarding patient history, the size and location of clusters (structures) with specialized-like cells in each heart (transverse IAS sections).

**Supporting table S**1. Data obtained on interatrial septum transverse sections.

| # | Sex | Age, years | Heart weight, g | Diagnosis | AF history | FO height, mm | FO width, mm | Specialized-like cells aggregated into a cluster (structure) | Structure height, mm | Structure length, mm | Structure thickness, mm | Structure volume, mm^3^ | Distance between the structure and AV node, mm | Structure localized in anterior FO | Structure localized in superior FO |
| --- | --- | --- | --- | --- | --- | --- | --- | --- | --- | --- | --- | --- | --- | --- | --- |
| 1 | m | 25 | 370 | w/o pathology | no | 19 | 17 | yes | 2 | 5 | 0,3 | 3,0 | 13 | 1 | 0 |
| 2 | m | 31 | 360 | w/o pathology | no | 13 | 12 | yes | 3 | 7 | 1,0 | 21,0 | 5 | 1 | 0 |
| 3 | m | 66 | 400 | w/o pathology | no | 20 | 20 | yes | 3 | 10 | 0,3 | 9,0 | 0 | 0 | 1 |
| 4 | m | 71 | 430 | CAD, MI | no | 9 | 10 | yes | 8 | 8 | 0,3 | 19,2 | 10 | 1 | 1 |
| 5 | f | 69 | 480 | CAD, MI | permanent | 27 | 27 | yes | 11 | 11 | 0,5 | 60,5 | 18 | 1 | 0 |
| 6 | m | 58 | 445 | CAD, MI | permanent | 22 | 17 | yes | 6 | 5 | 0,5 | 15,0 | 7 | 1 | 0 |
| 7 | m | 50 | 405 | CAD, MI | no | 9 | 10 | yes | 3 | 10 | 0,6 | 18,0 | 40 | 0 | 1 |
| 8 | f | 55 | 440 | CAD, MI | no | 28 | 27 | yes | 3 | 12 | 0,1 | 3,6 | 20 | 1 | 1 |
| 9 | m | 50 | 463 | CAD, MI | permanent | 8 | 10 | yes | 8 | 4 | 0,1 | 3,2 | 35 | 1 | 1 |
| 10 | m | 49 | 489 | CAD, MI | permanent | 6 | 10 | yes | 5 | 7 | 0,1 | 3,5 | 40 | 0 | 1 |
| 11 | f | 63 | 420 | CAD, MI | no | 13 | 13 | yes | 6 | 5 | 0,5 | 15,0 | 0 | 1 | 1 |
| 12 | m | 62 | 420 | CAD, MI | no | 22 | 22 | yes | 15 | 6 | 0,3 | 27,0 | 14 | 1 | 0 |
| 13 | m | 64 | 445 | CAD, MI | paroxysmal | 17 | 15 | yes | 13 | 2 | 1,0 | 26,0 | 27 | 1 | 0 |
| 14 | m | 76 | 520 | CAD, MI | permanent | 21 | 18 | yes | 20 | 5 | 0,5 | 50,0 | 5 | 1 | 0 |
| 15 | f | 81 | 450 | CAD, MI | paroxysmal | 22 | 20 | yes | 23 | 12 | 0,3 | 82,8 | 19 | 1 | 1 |
| 16 | f | 68 | 590 | Aortic stenosis | no | 21 | 22 | yes | Stucture borders not clear | Stucture borders not clear | Stucture borders not clear | Stucture borders not clear | Stucture borders not clear | 1 | 1 |
| 17 | m | 48 | 550 | Aortic stenosis | no | 26 | 15 | yes | 9 | 6 | 0,3 | 16,2 | 35 | 1 | 1 |
| 18 | f | 62 | 510 | Rheumatic mitral valve | permanent | 31 | 30 | yes | 31 | 12 | 0,4 | 148,8 | 19 | 1 | 0 |
| 19 | f | 59 | 560 | Rheumatic mitral valve | no | 7 | 10 | yes | Stucture borders not clear | Stucture borders not clear | Stucture borders not clear | Stucture borders not clear | Stucture borders not clear | 1 | 0 |
| 20 | m | 50 | 315 | Dilated cardiomyopathy | no | 17 | 13 | yes | 8 | 7 | 0,5 | 28,0 | 5 | 0 | 1 |
| 21 | m | 44 | 350 | CAD, MI | no | 11 | 15 | yes | 10 | 13 | 0,5 | 65,0 | 7 | 1 | 0 |
| 22 | f | 64 | 370 | GI cancer; w/o cardiac pathology | no | 19 | 15 | yes | 23 | 5 | 0,5 | 57,5 | 0 | 1 | 1 |
| 23 | f | 65 | 460 | GI cancer; LV hypertrophy | no | 7 | 15 | yes | 5 | 3 | 0,3 | 4,5 | 5 | 1 | 0 |
| **Mean** | 9 f (39%) | 57.8±13.3 | 445.3±70.2 | 3 (17%) w/o cardiac pathology | 8 (35%) | 17.2±7.4 | 16.7±5.9 | 100% | 10.2±8.0 | 7.4±3.3 | 0.4±0.2 | 32.2±35.4 | 15.4±13.2 |  |  |

AF, atrial fibrillation; AV node, atrio-ventricular node; CAD, coronary artery disease; FO, fossa ovalis; GI, gastrointestinal; F, female; M, male; LV, left ventricle; MI, myocardial infarction; w/o pathology, without pathology (no structural disease was found).
