# Supplementary material for: Evidence of Specialized Tissue in Human Interatrial Septum: Histological, Immunohistochemical and Ultrastructural Findings
Source: PLoS One. 2014 Nov 20;9(11):e113343. doi: 10.1371/journal.pone.0113343 (PMC4239074; doi:10.1371/journal.pone.0113343)
Supplement: Table S2 — Data obtained on interatrial septum longitudinal sections. (DOC) [file pone.0113343.s006.doc]

**Supporting TABLE S2**

Individual data regarding patient history and the location of clusters (structures) with specialized-like cells in each heart (longitudinal IAS sections).

**Supporting t**able S2. Data obtained on interatrial septum longitudinal sections.

| # | Sex | Age, years | Diagnosis | AF history | Specialized-like cells aggregated into a cluster (structure) | Distance between RSPV and the structure, mm | Distance between RSPV and flap valve, mm |
| --- | --- | --- | --- | --- | --- | --- | --- |
| 1 | f | 77 | CAD, MI | paroxysmal | yes | 12 | 20 |
| 2 | m | 23 | w/o pathology | no | yes | 10 | 10 |
| 3 | f | 56 | Rheumatic mitral valve | permanent | yes | 10 | 30 |
| 4 | f | 66 | CAD, MI | no | yes | 12 | 20 |
| 5 | m | 72 | CAD, MI | paroxysmal | yes | 12 | 25 |
| 6 | m | 67 | CAD, MI | no | yes | 10 | 20 |
| 7 | f | 28 | w/o pathology | no | yes | 9 | 17 |
| 8 | m | 75 | GI cancer | no | yes | 11 | 25 |
| 9 | m | 53 | Rheumatic mitral valve | paroxysmal | yes | 10 | 30 |
| 10 | m | 74 | CAD, MI | paroxysmal | yes | 13 | 25 |
| 11 | m | 62 | CAD, MI | paroxysmal | yes | 8 | 25 |
| 12 | f | 59 | GI cancer | paroxysmal | yes | 12 | 20 |
| 13 | m | 46 | Dilated cardiomyopathy | permanent | yes | 11 | 35 |
| 14 | m | 55 | Dilated cardiomyopathy | permanent | yes | 35 | 27 |
| 15 | m | 67 | CAD, MI | no | yes | 14 | 25 |
| 16 | m | 31 | w/o pathology | no | yes | 11 | 20 |
| 17 | m | 60 | CAD, MI | no | yes | 12 | 27 |
| **Mean** | 5 f (29%) | 57.1±16.5 | 3 (18%) w/o pathology | 9 (53%) | 100% | 12.5±5.9 | 23.6±5.8 |

AF, atrial fibrillation; F, female; M, male; CAD, coronary artery disease; GI, gastrointestinal; MI, myocardial infarction; RSPV, right superior pulmonary vein ostium; w/o pathology, without pathology (no structural disease was found).
